# Cassava molecular genetics and genomics for enhanced resistance to diseases and pests

**DOI:** 10.1111/mpp.13402

**Published:** 2023-11-07

**Authors:** Valentine Otang Ntui, Jaindra Nath Tripathi, Samwel Muiruri Kariuki, Leena Tripathi

**Affiliations:** ^1^ International Institute of Tropical Agriculture Nairobi Kenya; ^2^ Department of Plant Sciences Kenyatta University Nairobi Kenya

**Keywords:** cassava, cassava brown streak disease (CBSD), cassava mosaic disease (CMD), genome editing, genomics‐assisted breeding, *Manihot esculenta*, new breeding techniques, RNAi

## Abstract

Cassava (*Manihot esculenta*) is one of the most important sources of dietary calories in the tropics, playing a central role in food and economic security for smallholder farmers. Cassava production is highly constrained by several pests and diseases, mostly cassava mosaic disease (CMD) and cassava brown streak disease (CBSD). These diseases cause significant yield losses, affecting food security and the livelihoods of smallholder farmers. Developing resistant varieties is a good way of increasing cassava productivity. Although some levels of resistance have been developed for some of these diseases, there is observed breakdown in resistance for some diseases, such as CMD. A frequent re‐evaluation of existing disease resistance traits is required to make sure they are still able to withstand the pressure associated with pest and pathogen evolution. Modern breeding approaches such as genomic‐assisted selection in addition to biotechnology techniques like classical genetic engineering or genome editing can accelerate the development of pest‐ and disease‐resistant cassava varieties. This article summarizes current developments and discusses the potential of using molecular genetics and genomics to produce cassava varieties resistant to diseases and pests.

## Graphical abstract

Combating cassava diseases and pests requires a holistic approach that combines traditional breeding methods, genomics and biotechnology innovations such as conventional genetic engineering and genome editing.

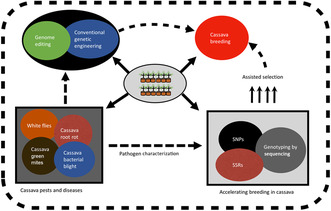

## 1 INTRODUCTION

**FIGURE 1:**
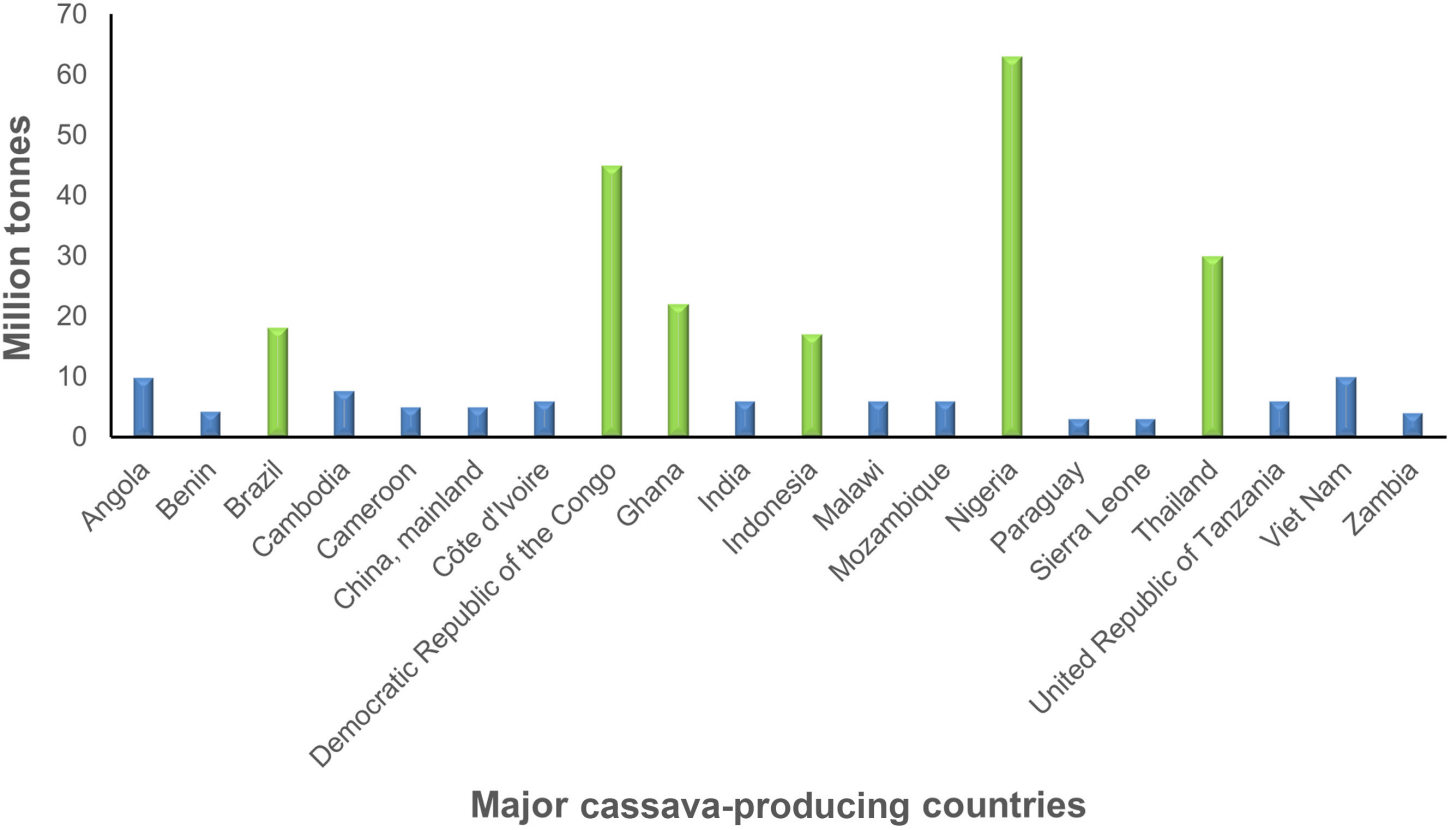
Major cassava production areas in the world. The green bars represent the top six cassava‐producing countries.

**FIGURE 2:**
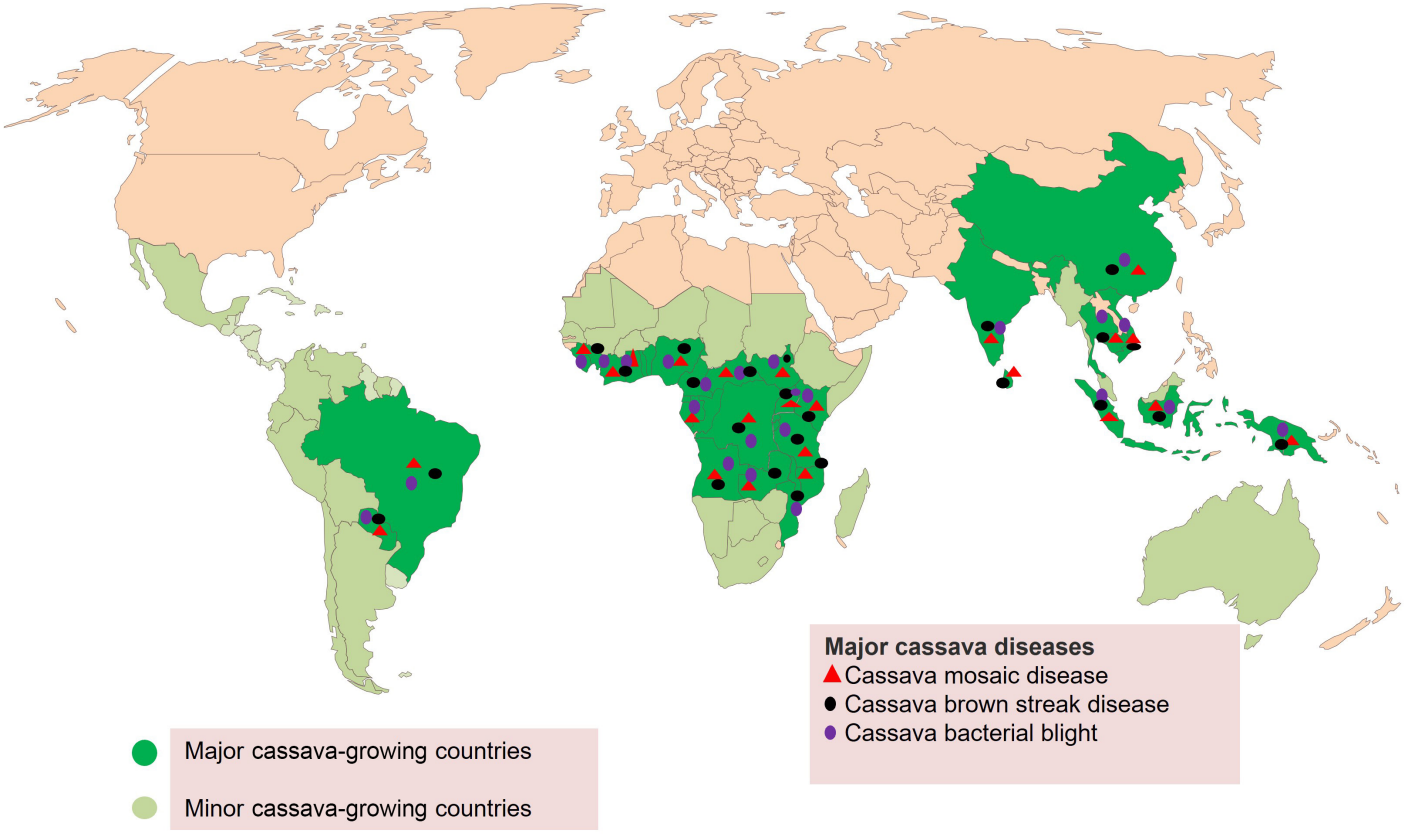
Cassava‐producing countries and the distribution of major casava diseases: cassava mosaic disease, cassava brown streak disease and cassava bacterial blight.

Cassava (*Manihot esculenta*) is an important tropical crop with annual production of about 315 million tonnes in over 30 million hectares. Africa accounts for 65% of the total global cassava production, with Nigeria being one of the major producers, contributing up to 20% the global total (FAOSTAT, 2022). Other major cassava growers are Angola, Brazil, China, the Democratic Republic of the Congo, Ghana, Indonesia, Mozambique, Vietnam and Thailand (Figures 1 and 2) (FAOSTAT, 2022).

Cassava is rich in carbohydrates and can be grown in poor soil conditions, making it a valuable source of food and income for more than 800 million people in rural areas (Nassar & Ortiz, 2010). It is a major source of staple food for human consumption after rice, maize, wheat and potato, and is also used as animal feeds and for production of commercial starch and biodegradable plastics. Cassava is a flowering hardy perennial shrub native to South American countries, domesticated about 8000 years ago and brought by Portuguese traders to west African countries during the sixteenth century. It is related to the *Euphorbiaceae* family and genus *Manihot*, which comprises about 98 species extending from shrubs to tree‐like relatives, including *Manihot glaziovii* for rubber production (Léotard et al., 2009; Nassar, 2008; Olsen & Schaal, 2007). It is a heterozygous crop species with 2n = 36 chromosomes and mostly polyploid in nature, cultivated as an annual crop in tropical and subtropical countries for its edible tuberous roots (El‐Sharkawy, 1993).

Cassava is a hardy crop that can be grown in a variety of conditions, making it an important crop for smallholder farmers who often have limited access to resources such as irrigation, fertilizers and pesticides. It is one of the most drought‐tolerant crops, growing on marginal nutrition‐depleted soils of acidic nature, with low production cost and the ability to survive in any environments. Cassava is extensively cultivated within latitudes 30° north and south of the equator, at 1500–2000 m a.s.l., in a temperature range of 25–29°C, with rainfall from 1000 to 1500 mm annually in the poor marginal soils (Onwueme & Sinha, 1991). Cassava is not severely affected by drought, but the changing climate can have a tremendous impact on cassava production due to the evolution of pathogens and pests, as well as extreme temperatures. Cassava production is already greatly hindered by diseases such as cassava mosaic disease (CMD) and cassava brown streak disease (CBSD), the two major viral diseases of cassava (Figure 2).

The crop is vegetatively propagated through stem cuttings ranging from 5000 to 20,000 cuttings per hectare, depending on the cropping system and purpose (Keating et al., 1988). However, this practice can also be a major source of spreading viral diseases such as CMD and CBSD. These viral diseases can cause significant yield losses, affecting food security and the livelihoods of smallholder farmers. To address this challenge, there is a need to develop disease‐resistant cassava varieties that can withstand the changing climate and resist viral diseases. In recent years, advances in molecular genetics and genomics have provided new tools and techniques for the development of improved cassava varieties. For instance, researchers have used RNA interference (RNAi) technology to control viral diseases. In addition, modern breeding approaches such as marker‐assisted selection and genome editing can accelerate the development of disease‐resistant cassava varieties. These techniques can help to identify and select desirable traits such as disease resistance, and rapidly develop improved varieties with higher yields and better nutritional content. With the increasing human population, which is projected to reach 9.8 billion in 2050 and 11.2 billion by 2100, there is a pressing need to increase food production to feed the population. Because cassava is one of the main staple food crops for Africa and some tropical countries, there is a need to develop strategies that will increase cassava production under extreme climate.

Recent progress and prospective on the application of molecular genetics and genomics for developing new varieties of cassava that are resistant to diseases and pests, and improve yield, quality and nutritional value have been promising. This article presents an overview of recent progress and prospective on the application of molecular genetics and genomics for developing cassava varieties resistant to diseases and pests.

## 2 CASSAVA DISEASES AND PESTS

**FIGURE 3:**
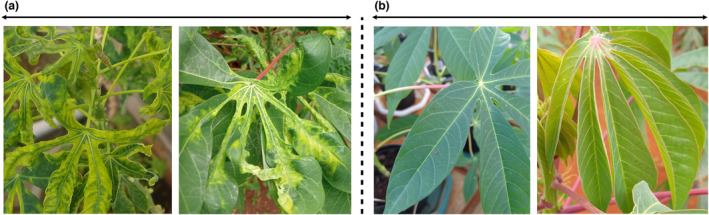
Cassava leaves: (a) leaves showing symptoms of cassava mosaic disease and (b) healthy leaves.

Cassava production is mainly constrained by two viral diseases, CMD and CBSD (Figure 2). These viruses are transmitted by insect vectors, mainly whitefly (*Bemisia tabaci*), and stem cuttings as planting materials (Legg et al., 2002). CMD is caused by viruses of the family *Geminiviridae* and genus *Begomovirus*. Yellow spots on newly opened leaves and retarded growth of the plant are typical symptoms of CMD‐infected cassava (Figure 3). CBSD is caused by two viruses, cassava brown streak virus (CBSV) and cassava brown streak Uganda virus (CBSUV) belonging to *Potyviridae* family and *Ipomovirus* genus (Mbanzibwa et al., 2009; Winter et al., 2010). The virus causes light yellowing and curling of leaves, retarded growth, stem lesions and severe necrosis in the roots, making them unacceptable for human consumption. These viral diseases and their vector whiteflies are prevalent throughout the growing season with varying severity, causing production losses up to 30%–50% (Patil & Fauquet, 2009). Cassava is propagated through stem cuttings and farmers obtain planting material from their own farms or surplus material from their neighbours. This practice leads to the accumulation and transmission of various pathogens, particularly viruses from the infected low‐quality planting material.

Cassava green mite (*Mononychellus tanajoa*), an indigenous pest, also causes symptoms similar to viral diseases and reduces crop yield in the infested field. The cassava green mite became established as a major pest of cassava across equatorial Africa in the early 1970s (Bellotti et al., 1999).

### 2.1 Cassava bacterial blight

Cassava bacterial blight disease (CBBD), caused by the bacterium *Xanthomonas axonopodis* pv. *manihotis*, is the most widespread bacterial disease of cassava (Katie et al., 2015). Since 1912, when it was first reported in Brazil, the disease has spread extensively to every part of the world where cassava is grown, including Asia, Africa and South America. The disease spreads from one area to another through infected cuttings, raindrops, use of contaminated farm tools, chewing insects and the movement of humans and animals through plantations, especially during or after rain.

The disease was first reported in Africa in Madagascar in 1946 (Livoi et al., 2018) and has now become a major constraint to cassava production (Figure 2), with yield losses of up to 100% reported in some countries (Livoi et al., 2018). Infected leaves exhibit angular, localized water‐soaked regions. Under severe disease conditions, there is significant defoliation, leaving bare stems referred to as candle sticks. The disease is systemic, with infected stems and roots exhibiting a brownish discolouration. Bacterial exudation (which appears as gum) can easily be seen on the bottom leaf surfaces of infected leaves as well as on the petioles and stems during times of high humidity. Wet weather is favourable for the development and spread of the disease. Management of the disease involves cultural practices, varietal resistance, biological control and sanitation practices. However, no durable broad‐spectrum resistance genes are available to breeders (Veley et al., 2023).

### 2.2 Cassava root rot disease

Cassava root rot disease (CRRD) is caused by a complex of soilborne pathogens and is one of the most destructive diseases of cassava, causing up to 100% yield losses in susceptible varieties (Hohenfeld et al., 2022). Several fungal species, including *Phytophthora drechsleri*, *Polyporus sulphureus*, *Fusarium oxysporum*, *Lasiodiplodia theobromae*, *Fusarium solani* and *Macrophomina phaseolina*, have been reported to cause CRRD (Akrofi et al., 2018). Environmental factors such as high temperatures, high humidity, waterlogging conditions in the soil and low soil fertility have been reported to promote CRRD (Akrofi et al., 2018). The disease symptoms include dark‐brown patches in the storage root tissue, wilting, browning and defoliation of the leaves. When the majority of the storage roots have decayed, the rot extends to the base of the plant and then there is lodging of the whole cassava plant. When only a few roots are affected, the cassava plants appear healthy and so the impact caused by the disease at this stage is not visible until harvested.

### 2.3 Cassava whiteflies

*B. tabaci* is a whitefly species complex that causes severe damage to cassava. *B. tabaci* causes direct damage to cassava by sucking plant sap and removing plant nutrients, thereby weakening the plants. Damage may be more severe when plants are under water stress. In addition, they excrete a sugary honeydew that serves as a food source for sooty moulds that inhibit photosynthesis and respiration (Nelson, 2008). The honeydew also contaminates the plant leaves, reducing their market value or eliminating their viability for sale. When *B. tabaci* infections are severe or prolonged, infested plants may wilt, turn yellow, become stunted or even die. *B. tabaci* is responsible for transmitting serious cassava viruses that cause CMD and CBSD, which in combination lead to significant yield loss (Maruthi et al., 2002).

## 3 BREEDING FOR RESISTANCE TO DISEASES AND PESTS

### 3.1 Breeding efforts for disease resistance improvement

In the major cassava‐growing regions, its production is limited by viral diseases (CBSD, CMD) and bacterial blight (CBB). This section summarizes the extensive efforts in breeding for improvement against these three major diseases. The resistance to CMD has been well characterized and was initially introgressed into *M. esculenta* from *M. glaziovii* in the late 1930s in Tanzania's Amani breeding centre (Jennings, 1957). Breeding efforts have progressed through the years in national research centres and at the Consultative Group on International Agricultural Research (CGIAR) centres, International Institute of Tropical Agriculture (IITA) and International Center for Tropical Agriculture (CIAT) (Lokko et al., 2007). Three types of CMD resistance exist in cassava: *CMD1*, which is controlled by a recessive gene; *CMD2*, a major dominant gene; and *CMD3*, which has *CMD2* in addition to a quantitative trait locus (QTL) (Utsumi et al., 2012). The CMD resistance present in the tropical *Manihot* species (TMS) series has been bred into other African genotypes (Jha et al., 2020; Turyagyenda et al., 2013; Utsumi et al., 2012). Resistant cultivars have also been identified in farmer‐preferred cultivars, exemplified by efforts in Benin and Nigeria (Utsumi et al., 2012; Xiao et al., 2019). Recently, there have been observations of breakdown in *CMD2* resistance when genotypes go through tissue culture (Li et al., 2017). The *CMD2* resistance is the dominant resistance, and these observations indicate why there is breakdown even as efforts to identify other sources of resistance are enhanced.

CBSD is the most devastating cassava disease in Africa (Collard et al., 2005). Unlike CMD, whose resistance breeding is well elaborated and implemented, CBSD tolerance is not as elaborately implemented mainly due to low levels of resistance, lack of appropriate CBSD characterization in resistant genotypes as well as genotype × environment interactions. The sources of resistance came from species *M. glaziovii* and *M. melanobasis* breeding efforts that began in Tanzania in the late 1950s and early 1960s. These early breeding efforts were entirely based on the polygenic inheritance nature of CBSD resistance. Efforts to characterize and map the resistance in farmer‐preferred cultivars in Tanzania and cultivars from other regions have resulted in the identification of loci in a number of these cultivars (Ruan et al., 2018; Wu et al., 2019). Due to complexity in the inheritance of CBSD resistance, efforts to characterize and map resistance will continue into the near future even as breeding efforts continue.

Efforts in breeding for resistance to CBB were initiated in early 1970s at IITA (Hahn et al., 1974). The resistance to CBB, just like CBSD, is polygenic, with regions of resistance identified in close proximity to *CMD2* (Rabbi et al., 2014). Mapping of sources of resistance in 150 lines from intraspecific crosses in cassava identified eight QTLs that could be used to assist in breeding (Jorge et al., 2001). However, the level of resistance linked to the eight QTLs was observed to have seasonal variations and additional sources of resistance may need to be mapped. Recent research has identified and characterized CBB resistance genes denoted as *MeLRR*s. Four *MeLRR*s were assessed on their ability to regulate resistance to *X. axonopodis* pv. *manihotis* infections in *Arabidopsis thaliana*. Despite the variations, the four *MeLRR*s were able to positively regulate disease resistance (Zhang et al., 2022). Such resistance genes could be bred into cassava or overexpressed through genetic engineering approaches.

There are mainly two major pests affecting cassava, whiteflies and mites, the former being the insect vector for two devastating cassava viruses, CBSV and CMV (Chalwe et al., 2015; Koros et al., 2018; Nukenine et al., 2002). In a study involving a 39‐year time‐series within which were major episodes of whitefly pandemics, increases in episodes of diseases transmitted by whiteflies were observed and these correlated with improved conditions of insect proliferation (Parry et al., 2020; Rabbi et al., 2014). Cassava breeding efforts for resistance to insect pests are limited because most of the focus has been on breeding for resistance to diseases (Houngue et al., 2019; Nzuki et al., 2017; Parry et al., 2020). There are African and South American genotypes that have been reported to have some level of resistance to whiteflies (Beyene et al., 2016; Ndunguru et al., 2016). Ten cassava genotypes evaluated in Uganda showed resistance to whitefly infestation and feeding damage, and it was concluded that these can be used as parental materials in breeding programmes for both whitefly and viral disease resistance (Beyene et al., 2016). In yet another study, a South American genotype MEcu72 and three Ugandan genotypes were observed to have resistance to whiteflies. In a similar study at the IITA in Nigeria, two genotypes, 96/1089A and TMS 30572, were observed to have supported the lowest number of whiteflies (Ariyo et al., 2005). These studies attest to the presence of resistance to whiteflies in genotypes from within the major cassava‐growing regions. These can be harnessed and integrated into breeding programmes within these regions for improved productivity.

Cassava green mites (CGM), unlike whiteflies, are vectors for a limited number of diseases, but are still devastating to cassava, resulting in up to 80% loss in productivity (Ezenwaka et al., 2018; Kayondo et al., 2018). They are especially destructive during the dry season (Praveen, 2018), which is of particular importance with projections that climate change will result in drier conditions. A substantial number of studies have identified resistance to mites in cassava genotypes (Kayondo et al., 2018; Masumba et al., 2017; Somo et al., 2020). Over 300 out of 5000 cultivars in the CIAT germplasm were observed to have some degree of resistance to CGM (Fang & Xiong, 2015). In another study at IITA Tanzania, 58 out of 377 clones tested under natural resistance to CGM were observed to have class I and II resistance (Shirima et al., 2020). Some of the clones from Zanzibar identified with CGM resistance were also observed to be high yielding and resistant to CMD, and had acceptable consumer quality (Shirima et al., 2020). The studies on resistance to CGM, just like in whiteflies, point towards possible breeding for their tolerance or resistance. Screening for tolerance to multiple pests has not been extensively reported and the possibility of having resistance to multiple pests (pyramiding) would not only enable an efficient way to use resources but would also save the time used in breeding.

### 3.2 Genetics and genomics characterization of resistance to cassava pathogens and pests

In recent years, molecular and genomics studies of pathogens and pests have been carried out to enhance the breeding of cassava for resistance (Ezenwaka et al., 2020). These studies have broadened the understanding of traits behind resistance and have also informed breeding approaches in different cassava‐growing regions. In this section, some of these studies and their applications to cassava improvement against the pests and diseases are explored.

The molecular basis for resistance to CMD is perhaps the best characterized of all the cassava diseases. Three regions that have been consistently associated with resistance to CMD were originally identified in the breeding programme in Amani, Tanzania (Ceballos et al., 2020). The first resistance region denoted *CMD1* is a recessive polygenic region that has been mapped using simple‐sequence repeat (SSR) markers (Mohan et al., 2013). *CMD1* was originally introgressed from the wild cassava relative *M. glaziovii* in Tanzania. The second, and most important, source of resistance (*CMD2*) is a major monogenic and dominant region identified in African landraces within a narrow geographical region in West Africa. *CMD2* has been mapped to chromosomes 8 and 12 using single‐nucleotide polymorphism (SNP) markers (Nzuki et al., 2017). This source of resistance has further been fine‐mapped using genotyping‐by‐sequencing, the results of which correlate with previous less‐detailed markers (Rabbi et al., 2014). There have been fears of instability of this resistance due to the narrow mode of its origin and the fast rate of cassava mosaic geminiviruses evolution. These fears are further exacerbated by recent observations that there is loss of *CMD2* resistance in plants that have undergone somatic embryogenesis in tissue culture (Beyene et al., 2016). Furthermore, two sequences enhancing geminivirus symptoms, and which are now considered to be satellite viruses, have also been observed to result in breakdown of *CMD2* resistance (Ndunguru et al., 2016). The third region of CMD resistance, *CMD3*, comprises two additive regions to *CMD2* that are thought to be epistatic in their mode of operation (Wolfe et al., 2016). To ensure sustained resistance, integrating the monogenic and polygenic sources of resistance has been proposed as a viable improvement approach (Rabbi et al., 2014). In the face of an evolving virus, the search for additional sources of resistance or integrating biotechnology techniques like genome editing to enhance the resistance could equally be viable options.

Resistance to CBSD has so far been found to be purely polygenic, unlike CMD (Shirima et al., 2020). The resistance for root necrosis and leaf symptoms seems to be in different chromosomes, as studies have consistently identified. In a cross between a Tanzanian landrace, Kiroba and the breeding line AR37‐80, for example, two QTLs associated with CBSD root necrosis and seven associated with leaf symptoms were mapped to chromosomes V and XII for the former and chromosomes IV, VI, XVII and XVIII for the latter (Nzuki et al., 2017). Similar QTLs, albeit in different chromosomes, were observed in crosses between the farmer‐preferred cultivars Namikonga and Albert (Masumba et al., 2017). The polygenic nature of these CBSD QTLs has further been corroborated in a study using SNP markers generated through genotyping‐by‐sequencing where two regions in chromosome 4 and 11 were linked to CBSD resistance (Kayondo et al., 2018). It is clear from these studies that CBSD resistance is still not as explored as CMD resistance. It is not clear, for example, the total as well as the individual contributions of these polygenic regions to CBSD resistance. There is therefore more effort needed in the identification of the roles of the different polygenic regions in CBSD resistance considering that this is the most devastating disease in cassava.

The basis for tolerance to CGM lies in traits like pubescent leaves, large compact shoot apices and improved leaf retention as well as stay green (Ezenwaka et al., 2018). It is thought that the natural predator of CGM, *Typhlodromalus aripo*, is attracted to leaf trichomes in pubescent leaves as they release volatile organic compounds (Ezenwaka et al., 2020). Two SSR markers (NS 1099 and NS 346) are strongly associated with CGM resistance (Choperena et al., 2012). In another study, two QTLs located on chromosomes V and X involved in CGM resistance were identified in F_1_ hybrids from crosses between Tanzanian landrace Kiroba and a breeding cultivar AR37‐80 (Nzuki et al., 2017). In a study using 42,204 SNP markers in F_1_ progenies of CGM‐resistant and ‐susceptible parents, one significant QTL (S12_7962234) located in chromosome 12 was identified (Ezenwaka et al., 2020). Nine novel genes were further linked to this QTL. In more recent studies, genome‐wide targeted approaches have been used to map CGM resistance. A genome‐wide association study to CGM resistance using a panel of 845 advanced breeding lines identified 35 SNP markers associated with CGM resistance and leaf pubescence in chromosome 8 (Ezenwaka et al., 2018). This genome‐wide study identified 17 genes linked to the CGM resistance in chromosome 8. Efforts should be geared towards identification of the exact mechanisms involved in resistance in QTL and SNPs in chromosome 8.

## 4 MODERN BIOTECHNOLOGICAL TOOLS FOR ENHANCING RESISTANCE TO DISEASES AND PESTS

**FIGURE 4:**
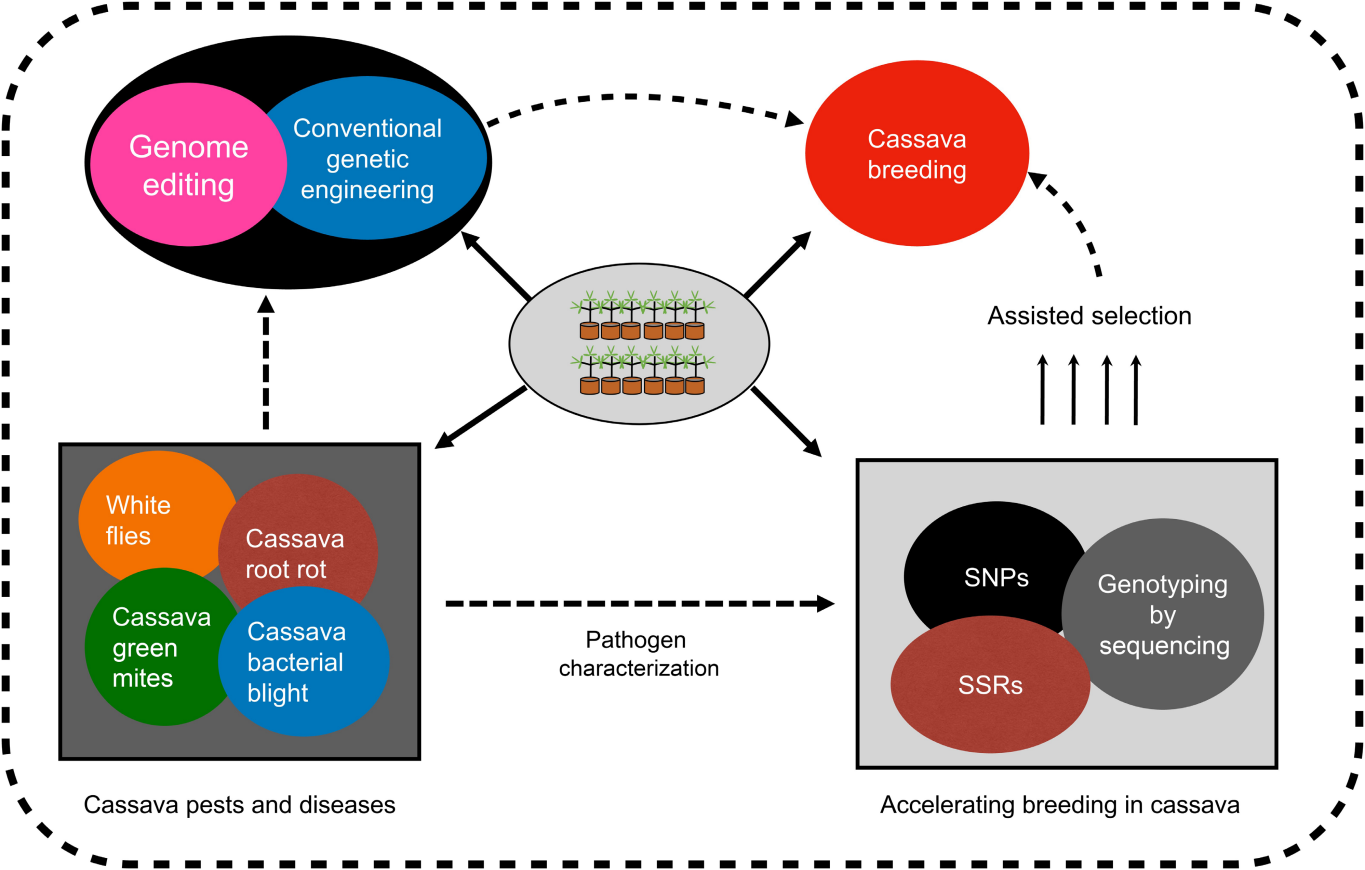
Schematic representation of the main approaches used for producing pest‐ and disease‐resistant cassava. The approaches involve using genomics to characterize cassava genotypes to identify diseases. Conventional genetic engineering and genome editing can be used to develop resistance to diseases and pests. The resistant cultivars can then be incorporated into breeding programmes. SNPs, single‐nucleotide polymorphisms; SSRs, simple‐sequence repeats.

Conventional breeding strategies to develop cassava for tolerance to pests and diseases is a major challenge because of the crop's poor flowering nature, polyploidy, vegetative propagation and heterozygosity, which prevent the transfer of desirable agronomic traits into the crop. To overcome these challenges, conventional breeding should be complemented with genetic engineering and genome‐editing techniques (Figure 4) to develop disease‐ and pest‐resistant cassava. In this section we will discuss the genetic engineering and genome editing strategies available for producing pest‐ and disease‐tolerant cassava.

### 4.1 Improvement of cassava for resilience to diseases by genetic engineering

The RNAi‐based silencing mechanism has been the main genetic engineering tool used to generate high‐level resistance to both CMD and CBSD (Chavarriaga‐Aguirre et al., 2016). RNAi, or posttranscriptional gene silencing, was developed as a mechanism for effective control of pathogens in crops (Susi et al., 2004). During the last two decades, RNAi has gained significant prominence as the method of choice for researchers to engineer pathogen resistance in crops. RNAi has an advantage over overexpression as it regulates gene expression via mRNA degradation, translation repression and chromatin remodelling through small noncoding RNAs, targets endogenous as well as exogenous genes and can be used in a highly targeted tissue‐specific manner to combat pathogens.

To engineer disease resistance by RNAi, a construct is designed such that when the transgene is expressed in the plant, it produces double‐stranded RNAs (dsRNAs). These dsRNAs are further processed by a Dicer (ds‐specific ribonuclease) to short interference RNA (siRNA) of 21–25 bp long. The siRNAs are then transported to a RNA‐induced silencing complex (RISC) where they are bound to the Argonaute proteins, which recognize and degrade homologous mRNA (Bonfim et al., 2007; Susi et al., 2004). The transgene can either target the plant host factors or the pathogen genes. Most RNAi studies on disease resistance are based on pathogen‐derived resistance, in which plants are transformed with genes or sequences derived from the pathogen to block the expression of the specific gene in the pathogen. For example, most existing cases of genetically engineered crops with resistance to viral pathogens via RNAi have targeted the coat protein, the movement protein or the replication protein of the virus.

The RNAi‐mediated resistance to CMD and CBSD mostly involved posttranscriptional silencing of *coat protein* (*CP*) genes, or the *AC1* (*Rep*), *AC2* (*TrAP*) or *AC3* (*REn*) genes implicated in the replication of viral DNAs (Chavarriaga‐Aguirre et al., 2016). The first report highlighting the use of RNAi to develop resistance to CMD was demonstrated by Zhang et al. (2005) using the easy‐to‐transform model cultivar TMS60444. The authors developed transgenic plants with increased resistance to African cassava mosaic virus (ACMV) by antisense RNA technology targeting the viral mRNAs of *Rep* (*AC1*), *TrAP* (*AC2*) and *REn* (*AC3*). Viral DNA replication assays in detached leaves as well as ACMV infection in transgenic plants showed strong reduction or inhibition of the replication of two isolates of ACMV in most of the transgenic lines (Zhang et al., 2005). Thereafter, Vanderschuren et al. (2007) introduced resistance to ACMV using an RNAi construct targeting the common region of ACMV DNA‐A into the cultivar TMS60444. Similarly, Vanderschuren et al. (2009) engineered transgenic TMS60444 with resistance to ACMV by expressing ACMV AC1‐homologous hairpin dsRNAs. Transgenic lines accumulated high levels of AC1‐homologous small RNAs and exhibited ACMV immunity under high viral pressure. Kasetsart University 50 (KU50), an elite cultivar widely grown by many farmers in Asia for its high dry matter content, is highly susceptible to CMD caused by the Sri Lankan cassava mosaic virus (SLCMV) (Dutt et al., 2005). Ntui et al. (2015) engineered KU50 cultivar for resistance to SLCMV by RNAi‐mediated silencing of the AV1 coat protein and AV2 pre‐coat protein genes. Transgenic lines accumulated high levels of siRNA and displayed increased resistance to SLCMV, and no virus load could be detected in uninoculated new leaves of the infected resistant lines.

RNAi technology was shown to generate high‐level resistance to CBSD, a major cassava disease in East Africa (Vanderschuren et al., 2012; Yadav et al., 2011). Transgenic TMS60444 lines expressing inverted‐repeat constructs of highly conserved sequences of CBSV and CBSUV of the viral CP (Vanderschuren et al., 2012) or full‐length coat protein (FL‐CP) of CBSUV (Yadav et al., 2011) demonstrated increased resistance to CBSD after viral inoculation. Due to immunity against CBSD, Vanderschuren et al. (2012) adapted this technology to generate transgenic cassava lines combining resistance to both CBSD and CMD. They expressed the hairpin construct in a CMD‐resistant farmer‐preferred Nigerian landrace TME 7 (Oko‐Iyawo). All transgenic TME 7 lines showed immunity against CBSV and CBSUV infections. The resistance was maintained when plants were co‐inoculated with East African cassava mosaic virus (EACMV), a geminivirus causing CMD.

The RNAi technology described above represents an important approach to tackling the problem of CMD and CBSD. Unfortunately, loss of CMD resistance in these transgenic plants has been reported in the field. Although the cause of such loss is unknown, it is hypothesized to be epigenetic regulation of the *CMD2* locus during the production of transgenic and non‐transgenic cassava plants through somatic embryogenesis. These findings indicate that cassava breeders need to look for alternative sources of CMD resistance in cassava, probably the use of cytokinin *meta*‐topolin [6‐(3‐hydroxybenzylamino) purine] to induce in vitro shoots on non‐embryogenic explants (Chauhan & Taylor, 2018), or work with *CMD1* and/or *CMD3* resistant cultivars.

Success in obtaining resistance to CBSV using RNAi technology has been achieved. Cassava with resistance to CBSD using RNAi technology has undergone field trials in Uganda and Kenyan, and very high levels of resistance were demonstrated (Wagaba et al., 2017). In 2021, Kenya approved CBSD‐resistant cassava for environmental release. The approval is a significant step to getting disease‐resistant cassava into the hands of Kenyan farmers to address food security challenges (NBA [National Biosafety Authority], Kenya, 2021).

Controlling the whitefly, which is a vector for CMVs and CBSV, would represent an alternative for the management of CMD and CBSD. The use of transgenic technology to control lepidopteran pests in maize, soybean, canola and cotton (James, 2015) has paved the way for controlling insect pests in crops. Although genetic engineering approaches have not been used to control whitefly and other insect pests of cassava, several insect genes have been identified that could be targeted for silencing in whitefly (Chavarriaga‐Aguirre et al., 2016; Jekayinoluwa et al., 2020).

In nymphs and adults of *Aleurotrachelus socialis* feeding on the whitefly‐resistant cassava landrace Ecu72, several differentially expressed genes have been identified (reviewed in Jekayinoluwa et al., 2020). Among the up‐regulated genes were chitinases, lipoxygenases, LOX5 and methyl‐transferases such as cafeoyl‐CoA‐o‐methyltransferase. Cafeoyl‐CoA‐o‐methyltransferase gene is involved in lignin synthesis. Given that salicylic acid, jasmonic acid and ethylene control several of the cellular biochemical paths that respond to pathogens and pests, the overexpression and/or down‐regulation of genes such as LOX5 or cafeoyl‐CoA‐o‐methyltransferase in cassava could be an important pathway for controlling whitefly.

Developmental genes play important roles in insect development and function. During insect development, several genes are either expressed or down‐regulated at different stages of the insect's lifecycle (Jekayinoluwa et al., 2020). Overexpression or down‐regulation of the genes throughout the insect's lifecycle could interfere with the insect's development and hence reduce virus transmission. For example, silencing the midgut gene *Rack1* in green peach aphid reduced the growth of gut cells and subsequently decreased nutrient uptake (Pitino et al., 2011). The salivary enzyme alkaline phosphatase, first identified in the saliva of a Russian wheat aphid, plays a major role in the penetration and feeding mechanism of the insect (Cooper et al., 2011). Blockage in the expression of salivary sheath protein (*shp*) required for the ingestion of phloem sap by RNAi led to decreased growth, fecundity and survival of grain aphid (Abdellatef et al., 2015).

Flight is an important activity that allows insect vectors to transmit diseases from plant to plant. During flight, essential genes such as 3‐ketoacyl‐CoA thiolase, phosphoenolpyruvate carboxykinase and glycogen phosphorylase‐like isoform 2, involved in lipid metabolism, increase in wing muscles. RNAi‐mediated silencing of such genes in citrus aphid impacted wing development (Shang et al., 2016). In *Aphis gossypii*, the abnormal wing disk (*awd1* and *awd2*) genes play a significant role in the development and differentiation of the insect. The genes encode a nucleoside diphosphate kinase and were reported to be significantly up‐regulated in wingless than in winged morphs (Yang et al., 2014). Loss of function of *awd* in *Drosophila* caused lethality (Yang et al., 2014), indicating the significance of these genes in the mobility and dispersal of insects. In green peach aphid, RNAi‐mediated silencing of the *acetylcholinesterase* (*MpAChE2*) gene, a serine hydrolase that regulates acetylcholine in insects, birds and mammals, resulted in resistance of transgenic plants to aphids (Guo et al., 2014a; Guo, Wang, et al., 2014). The implication of these studies is that these genes could be silenced in whitefly to interfere with the feeding mechanism, movement and development of the insect, leading to death and hence reduced CMV and CBSV transmission.

### 4.2 Genome‐editing strategies to develop disease‐resistant cassava

In recent years, genome editing, the ability to perform controlled/precise changes in the genome of an organism using specific nucleases, has become the tool of choice to modify plants. Several nucleases, including meganucleases, zinc finger nucleases (ZFNs), transcription activator‐like effector nucleases (TALENs) and the clustered regularly interspaced short palindromic repeats (CRISPR)/Cas associate protein (Cas) (CRISPR/Cas), have been developed to achieve effective genome editing in organisms (Tripathi et al., 2019). Among these nucleases, CRISPR/Cas9, which was developed from the adaptive immunity system of *Streptococcus pyogenes*, is widely used as the most effective genome‐editing tool in plants because of its simplicity, design flexibility, high efficiency and ability to edit multiple genes simultaneously (multiplexing) (Ntui et al., 2020; Tripathi et al., 2019).

The CRISPR/Cas9 technology comprises two basic components: the Cas9 nuclease and the gRNA (guide RNA). The Cas9 recognizes target DNA by gRNA–DNA pairing between the 5′ leading sequence of gRNA and the target sequence. It also recognizes the protospacer adjacent motif (PAM) sequence and starts editing upstream of the sequence. The PAM is a three‐nucleotide sequence, usually NGG or NAG, where N can be any nucleotide base, and serves as a recognition segment for Cas9 to start editing upstream. Usually, Cas9 shows more affinity to NGG than NAG. The gRNA directs the endonuclease Cas9 to induce precise double‐stranded break (DSB) cleavage at a target site; repair of this site by the cell's own natural repair mechanism of homology‐directed repair (HDR) or nonhomologous end joining (NHEJ) can produce a user‐desired mutation or genetic outcome. The NHEJ repair, which is error prone, creates random insertions and deletions (SNPs, indels) and results in frameshift mutations and targeted gene knockouts. The HDR pathway is more precise in the repair of a DNA sequence, leading to gene knock‐in, gene replacement or insertion of foreign genes or DNA sequences. Based on the type of repair, the editing is classified into three types: SDN1, SDN2 or SDN3 (Modrzejewski et al., 2019). SDN1, which is based on NHEJ results in random mutations in the host genome, causes gene silencing, gene knock‐out or alteration in the gene function. When the repair template identical to the DSB is added and the repair is via HDR, resulting in nucleotide substitution or targeted indels, it is referred to as SDN2. In SDN3, the DSB is repaired via HDR using a repair template that is longer than the homologous sequences in which the DSB is made, resulting in the targeted insertion of foreign genes. The ability of CRISPR/Cas9 to create DSBs at sequence‐specific targets in the DNA or RNA molecules makes it an excellent tool to engineer disease and pest resistance in crops. Recently, CRISPR/Cas variants with different editing strategies, such as Cas12a (Cpf1), Cas13, CRISPR activation (CRISPRa), CRISPR interference (CRISPRi), base editing and prime editing, have been developed and used for editing in plants (Joshi et al., 2020; Tripathi et al., 2022).

The first report on CRISPR/Cas9 editing of cassava was established using the *phytoene desaturase* (*PDS*) gene (Odipio et al., 2017). PDS is an enzyme in the carotenoid biosynthesis pathway involved in the conversion of colourless phytoene into lycopene, a coloured compound in the pathway (Kaur et al., 2018). Knockout of *PDS* usually results in the production of albino phenotypes (Kaur et al., 2018; Ntui et al., 2020). Odipio et al. (2017) edited *PDS* in the model cultivars TMS60444 and TME 204, and obtained a mutation frequency of 90%–100%, with most of the plants exhibiting an albino phenotype. Their report opened avenues for genome editing of cassava for resistance to CMD and CBSD.

Gomez et al. (2019) generated transgenic cassava plants expressing Cas9 and gRNAs targeting *nCBP‐1* and *nCBP‐2*, which are isoforms of *eIF4E*. The eukaryotic translation initiation factor (*eIF*) gene family, including eIF4E and its paralogue eIF(iso)4E, also known as cap‐binding protein, are essential susceptibility genes required for the cellular infection cycle of potyviruses, which have single‐stranded, positive‐sense RNA (ssRNA+) genomes. In plants, some host genes classified as susceptibility genes (*S* genes) facilitate pathogen invasion and thus are considered essential for compatible plant–pathogen interactions (Zaidi et al., 2020). During pathogen invasion, these genes are activated by the pathogen to favour pathogen growth and promote symptom development. Editing of *S* genes has been reported to confer resistance to the corresponding pathogen and, in some cases, broad‐spectrum resistance (Kim et al., 2018; Peng et al., 2017). Transgenic cassava plants generated by editing the susceptibility gene *eIF4E* (*nCBP‐1* and *nCBP‐2*) exhibited partial resistance against CBSD (Gomez et al., 2019). Other susceptibility genes, such as *mildew resistance locus O* (*MLO*) (Wang et al., 2014), ERF transcription factor, *WRKY* and *MYB*, *PMR4* (Santillán Martínez et al., 2020), Sugar Will Eventually be Exported Transporters (*SWEET*) (Oliva et al., 2019), *Lateral Organ Boundaries* (*CsLOB1*) (Jia et al., 2016; Peng et al., 2017), as well as fungal receptor genes such as downy mildew resistance 6 (*DMR6*) (Tripathi et al., 2021) or their promoters can be edited in cassava to develop resistance to other pathogens.

Editing of viral genes is an important strategy to develop resistance against viruses. DNA as well as RNA viral genes have successfully been edited by Cas9, Cas12a or Cas13a (Aman et al., 2018; Kalinina et al., 2020; Khatodia et al., 2017; Tripathi et al., 2019; Yin & Qiu, 2019; Zhang et al., 2019). Ali et al. (2015) were among the first to use CRISPR/Cas9 to induce resistance to viruses. They designed gRNAs targeting the viral Rep, coat protein and conserved intergenic region (IR) of tomato yellow leaf curl virus (TYLCV). The gRNAs were integrated to tobacco rattle virus (TRV) vector and delivered to *Nicotiana benthamiana* plants overexpressing Cas9 by agroinfiltration. *N. benthamiana* plants having mutations in the target sequences were resistant to TYLCV (Ali et al., 2015). Similarly, Ji et al. (2015) engineered *Arabidopsis* and *N. benthamiana* resistant to beet severe curly top virus. Editing of IR and CI coding regions in tobacco resulted in complete resistance to cotton leaf curl Multan virus (Yin et al., 2019). Later, *N. benthamiana* plants agroinfiltrated with a multiplexed CRISPR/Cas9 construct targeting the Rep, and *βC1* gene of the beta satellites exhibited delayed symptoms and lower virus titres of cotton leaf curl virus (CLCuV) (Khan et al., 2020). In contrast, editing of *AC2* and *AC3* genes, which encode the transcription activator protein and the replication enhancer protein of ACMV, respectively, failed to generate resistance against the virus in transgenic cassava (Mehta et al., 2019). Instead, the authors observed that CRISPR/Cas9 editing resulted in the formation and escape of new (CRISPR‐resistant) ACMV variants that were probably generated due to post‐cleavage repair. Altogether, this study showed that direct targeting of viral genes is a powerful tool for engineering resistance to viruses; however, in some cases, it may trigger rapid evolution of the virus, resulting in the development and release of new pathogenic virus forms, as reported by Mehta et al. (2019). In this regard, adequate knowledge of the roles of viral genes will be necessary to identify potential crucial genes for editing. One of the major devastating diseases of cassava is CBSD caused by RNA virus variants. The recent identification of the RNA‐only targeting Cas13 is a welcome addition to the genome‐editing arsenal that could directly benefit cassava improvement against RNA‐virus diseases such as CBSD. Cas13 has successfully been used to target plant viruses (Mahas et al., 2019). The presence of transcribed RNAs in DNA viruses also means that Cas13 could also be used to target not only RNA viruses but also transcribed DNA‐genome viruses such as CMV.

## 5 PATHOGEN EVOLUTION DYNAMICS AND CASSAVA IMPROVEMENT

Breakdown in resistance against CMV has already been reported (Ndunguru et al., 2016), a trend that is expected for other major cassava pathogens as they evolve. Integrating durable resistance against the major pests and diseases of cassava should therefore be informed by pathogen evolution and host–pathogen interactions. Constant surveillance of the major cassava pathogens to establish their evolutionary trajectories is an important way this can be achieved. Efforts have been going on to achieve pathogen evolution and surveillance, although at minimal scale. Minimal efforts to integrate pathogen evolution data to mainstream cassava improvement have been achieved so far. However, efforts to track diversity through surveillance of pathogens are underway. The CBSV and UCBSV evolution was assessed through genomics where two virus variants with different evolutionary trajectory but corresponding to UCBSD outbreaks were identified (Mbanzibwa et al., 2011). Additional studies using genomics have observed that CBSV has a faster genome evolution compared to UCBSV, a difference that was correlated with disease severity and host plant diversity (Alicai et al., 2016). Molecular and serological approaches have also been used to identify Sri Lanka cassava mosaic virus (SLCMV) variants in China (Wang et al., 2020). Surveillance has also identified co‐infections and pest resurgence within cassava farms in Congo (Bisimwa et al., 2019). SLCMV surveys have also been conducted in Thailand, with observations revealing a relatively high level of similarity to SLCMV (Saokham et al., 2021). A combination of surveillance and evolutionary analyses was able to identify a new cassava mosaic virus named African cassava mosaic Burkina Faso virus (ACMBFV), a recombinant between tomato leaf curl Cameroon virus and cotton leaf curl Gezira virus (Tiendrébéogo et al., 2012).

Pathogen evolution has been observed to result in enhanced pathogenicity and reduced tolerance/resistance in cassava. Modern breeding approaches that incorporate high‐throughput sequencing technologies, genetic engineering and genome‐editing technologies can be used to enhance resistance durability.

## 6 PAY‐OFF GAPS ASSOCIATED WITH MOLECULAR GENETIC TOOLS

Cassava yields in Africa are mostly small as a result of many factors, including abiotic, biotic and associated crop management practices. Biotic factors such as diseases and pests cause a wide gap between the expected yield and that which farmers achieve. However, this gap becomes smaller as farmers adopt better agronomic practices, including increasing fertilizer, irrigation or the use of pest‐ and disease‐resistant cultivars (Srivastava et al., 2023). The molecular genetics and genomics tools described here have the potential to reduce the yield gap by producing disease‐ and pest‐resistant cultivars. The deployment of resistant cultivars provides an efficient means to control disease and pests. However, the advantages of using the resistant variety may be outweighed by a yield penalty, in which case the susceptible variety outperforms the resistant one in the absence of disease or pest. The problem with this is that using the resistant variety is only advised if the disease manifests and is severe enough for the resistant variety to outperform the susceptible variety. The likelihood of a disease or pest invasion is decreased when the resistant variety is planted, which provides an additional benefit. As a result, from the viewpoint of a grower community, the cropping density for the resistant variety is likely to be at an ideal pay‐off (Vyska et al., 2016). Although there are not enough scientifically derived data to definitively assign yield estimates to the cassava improvement methods discussed here, and consequently their economic impact on cassava production, they are nonetheless important factors to closing the cassava yield gap. For example, when the potential for an outbreak is uncertain, planting the resistant variety reduces the probability of an outbreak occurring and hence lowers the yield gap.

## 7 FUTURE PERSPECTIVES

Currently, the method of choice for producing cassava with disease resistance through genome editing is by plasmid delivery of CRISPR/Cas reagents. This method is laborious, and many farmers' preferred genotypes are still recalcitrant to genetic transformation. Techniques like in planta transformation or agroinfiltration could be used. Platforms based on viral vectors could be used to deliver CRISPR/Cas9 constructs quickly and effectively. CMV could be modified to carry the CRISPR/Cas9 reagents into the plant genome through agroinfiltration. This method has been demonstrated in tomato and tobacco (Ali et al., 2015).

Production of DNA‐free genome‐edited cassava could boost its productivity and acceptability. Until now, cassava genome editing has been based on plasmid delivery of CRISPR/Cas9 reagents, which are integrated into the genome and thus the product is regulated as a genetically modified organism. This may reduce its acceptability in many countries. Cassava is vegetatively propagated, hence transgenes cannot easily be eliminated by segregation as in sexually propagated crops, even with CRISP/Cas9‐mediated plasmid delivery. This will be a significant obstacle to creating cassava genotypes that are resistant to diseases and pests using genome editing. However, direct delivery of preassembled Cas9 protein‐gRNA ribonucleoproteins (RNPs) into cassava cells may be able to overcome these restrictions because the RNPs are rapidly broken down by indigenous proteases after being delivered, leaving no signs of foreign DNA elements behind. The mutant genotypes will not encounter any significant regulatory problems.

Recently, researchers have developed a CRISPR/Cas editing system in which grafting a wild‐type shoot to a transgenic donor rootstocks produced DNA‐free genome‐edited lines (Yang et al., 2023). In this system, a transgenic genome‐edited root is produced. On grafting the root with a wild‐type shoot, the Cas9 and the gRNA transcripts are transported from the transgenic rootstock to the shoot, where they create mutations in the shoots without integration. The resulting shots and seeds are foreign DNA‐free genome‐edited lines. Using this method, the stress of going through tissue culture is avoided. Cassava breeders/biotechnologists could explore the possibilities of using this method to produce foreign DNA‐free genome‐edited cassava. Grafting in cassava has already been demonstrated (Souza et al., 2018).

Despite being used minimally in plants, nanobody (Nb)‐mediated virus resistance is emerging as a new way of generating resistance. The stable overexpression of grapevine fanleaf virus‐specific Nbs in *N. benthamiana* and grapevine, for example, conferred resistance to the virus in both plants (Hemmer et al., 2018). The small sizes of Nbs could allow for their delivery through viral vectors and thus circumvent the need for a tissue culture process involved in cassava genetic engineering.

## 8 CONCLUSIONS

Cassava, which is a major food security crop for millions of people in Africa and other tropical regions, faces a challenge posed by viral diseases, which have remained the primary cause of the crop's low productivity. Observed breakdown in resistance for some traits, such as CMD resistance, requires a regular re‐evaluation of existing traits to ensure they are coping with the continued pressures. Innovative strategies such as gene pyramiding and diversification of resistance sources can provide effective options for enhancing cassava resilience to diseases. For instance, using genomic tools such as SSR, SNP genotyping by sequencing and modern biotechnologies such as genome editing and genetic engineering enable the prospect of modifying the existing genes in susceptible cassava varieties and conferring disease resistance, thereby revolutionizing cassava improvement efforts. A thorough understanding of the mode of action and potential target genes is necessary for the integration of biotechnology technologies such as traditional genetic engineering and genome editing. While significant progress has been made in identifying target genes and QTLs, more studies are required to fully elucidate the mode of action of these genes and their potential as targets for genome editing. Based primarily on data from other crops, several possible targets for genome editing in cassava have been described here. It is now necessary to identify the cassava homologues of these targets. Overall, the best method for combating cassava diseases and pests may be a holistic approach that combines traditional breeding methods and biotechnology innovations for cassava improvement to sustainably enhance cassava productivity and ensure food security for millions of people in the tropics.

## CONFLICT OF INTEREST STATEMENT

The authors declare no competing interests exist.

## Data Availability

Data sharing is not applicable to this article as no new data were created or analysed.
