# Correction to Cassava molecular genetics and genomics for enhanced resistance to diseases and pests

**DOI:** 10.1111/mpp.13432

**Published:** 2024-02-08

Ntui V.O., Tripathi J.N., Kariuki S. M., Tripathi L (2024) Cassava molecular genetics and genomics for enhanced resistance to diseases and pests. *Molecular Plant Pathology*, 25, e13402. https://doi.org/10.1111/mpp.13402

Cassava brown streak disease is limited to East Africa and some countries in Central and South Africa. However, in Figure 2 in the published version of this article, we mistakenly attributed it to other countries in West Africa, South America, Asia, and Oceania. This mistake happened during preparation of the figure. This figure error does not in any way change the conclusions of the manuscript. The corrected Figure 2 is shown below.

**FIGURE 2:**
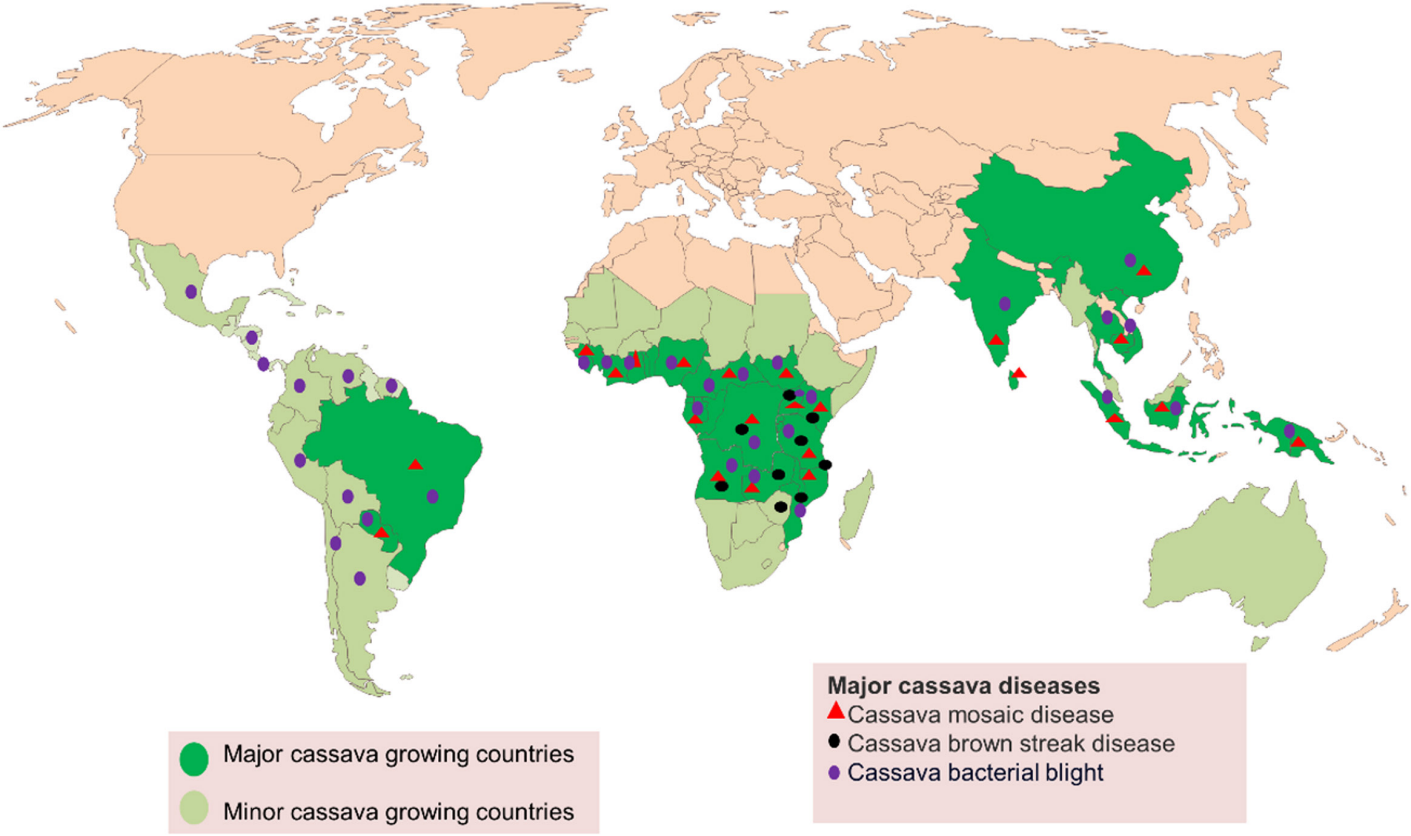
Cassava‐producing countries and the distribution of major casava diseases: cassava mosaic disease, cassava brown streak disease and cassava bacterial blight.

We apologize for this error.

